# Human tNeurons reveal aging-linked proteostasis deficits driving Alzheimer’s phenotypes

**DOI:** 10.21203/rs.3.rs-4407236/v1

**Published:** 2024-05-30

**Authors:** Ching-Chieh Chou, Ryan Vest, Miguel A. Prado, Joshua Wilson-Grady, Joao A. Paulo, Yohei Shibuya, Patricia Moran-Losada, Ting-Ting Lee, Jian Luo, Steven P. Gygi, Jeffery W. Kelly, Daniel Finley, Marius Wernig, Tony Wyss-Coray, Judith Frydman

**Affiliations:** 1 Department of Biology, Stanford University, Stanford, California, USA; 2 Department of Genetics, Stanford University, Stanford, California, USA; 3 Wu Tsai Neurosciences Institute, Stanford University School of Medicine, Stanford, California, USA; 4 Department of Chemical Engineering, Stanford University, Stanford, California, USA; 5 Department of Neurology and Neurological Sciences, Stanford University, Stanford, California, USA; 6 Qinotto, Inc. San Carlos, California, USA; 7 Department of Cell Biology, Harvard Medical School, Boston, Massachusetts, USA; 8 Instituto de Investigación Sanitaria del Principado de Asturias (ISPA), Oviedo, Spain; 9 Departments of Pathology, Stanford University School of Medicine, Stanford, California, USA; 10 Institute for Stem Cell Biology and Regenerative Medicine, Stanford University School of Medicine, Stanford, California, USA; 11 Palo Alto Veterans Institute for Research, Inc. (PAVIR), Palo Alto, California, USA; 12 Department of Chemistry, The Scripps Research Institute, La Jolla, California, USA.; 13 The Skaggs Institute for Chemical Biology, The Scripps Research Institute, La Jolla, California, USA.; 14 Aligning Science Across Parkinson’s (ASAP) Collaborative Research Network, Chevy Chase, MD, USA.; 15 Lead contact

## Abstract

Aging is a prominent risk factor for Alzheimer’s disease (AD), but the cellular mechanisms underlying neuronal phenotypes remain elusive. Both accumulation of amyloid plaques and neurofibrillary tangles in the brain^1^ and age-linked organelle deficits^2–7^ are proposed as causes of AD phenotypes but the relationship between these events is unclear. Here, we address this question using a transdifferentiated neuron (tNeuron) model directly from human dermal fibroblasts. Patient-derived tNeurons retain aging hallmarks and exhibit AD-linked deficits. Quantitative tNeuron proteomic analyses identify aging and AD-linked deficits in proteostasis and organelle homeostasis, particularly affecting endosome-lysosomal components. The proteostasis and lysosomal homeostasis deficits in aged tNeurons are exacerbated in sporadic and familial AD tNeurons, promoting constitutive lysosomal damage and defects in ESCRT-mediated repair. We find deficits in neuronal lysosomal homeostasis lead to inflammatory cytokine secretion, cell death and spontaneous development of Aß and phospho-Tau deposits. These proteotoxic inclusions co-localize with lysosomes and damage markers and resemble inclusions in brain tissue from AD patients and APP-transgenic mice. Supporting the centrality of lysosomal deficits driving AD phenotypes, lysosome-function enhancing compounds reduce AD-associated cytokine secretion and Aβ deposits. We conclude that proteostasis and organelle deficits are upstream initiating factors leading to neuronal aging and AD phenotypes.

Aging is a central contributing factor to Alzheimer’s disease (AD). Numerous observations in different model systems indicate that aging is linked to a decline in protein homeostasis (proteostasis) and organelle homeostasis^2,3^, including endosomal-lysosomal function^4–7^. Models genetically mimicking AD that accumulate misfolded protein aggregates, including Aβ and tau, in characteristic amyloid plaques and neurofibrillary tangles^1^ also have defects in endosome-lysosomal function and autophagic clearance^3,8–10^. The mechanistic underpinnings of these deficits, and how they relate to human brain aging and disease remain poorly understood. Because extra-cellular amyloids, such as Aβ and tau, can be taken up into neurons leading to lysosomal damage and cell death^11–14^, it is proposed that AD pathologies are largely mediated by non-cell autonomous effects. However, such hypothesis cannot account for early events leading to proteotoxic amyloid production. It is clear that understanding the molecular origins of pathologic processes in neurons of AD patients will be essential to uncover effective treatments.

Developing cellular systems to enable mechanistic study of proteostasis and organellar phenotypes caused by aging and AD in human neurons remains challenging. Human neurons, including from postmortem brains, are widely studied at single-cell transcriptomics^15^. However, changes in cytonuclear and organelle-specific proteostasis networks are often not apparent from these datasets. Another common tool is to derive induced pluripotent stem (iPS) cells from patient somatic cells and reprogram them to different neuronal lineages^16^. However, this process restores youthfulness to the induced neurons (iNeurons), forgoing the key contribution of aging to neurodegenerative phenotypes^16,17^. The recent development of approaches to transdifferentiate neurons (herein tNeurons) directly from human adult somatic cells enables studying neurodegenerative diseases while maintaining the contribution of aging^17,18^. Thus, tNeurons derived from various neurodegenerative disease patients retain aging epigenetic marks, exhibit disease phenotypes and have even led to potential therapeutic strategies^19–22^. Due to limiting samples, tNeurons have been primarily examined via RNA sequencing. To illuminate the proteome changes and cellular defects linked to aging and AD, we here improved neuronal transdifferentiation of human adult somatic cells. We derived tNeurons from dermal fibroblasts obtained from a panel of healthy young and aged donors, as well as donors with sporadic AD (sAD) and familiar AD (fAD). Using quantitative proteomic analyses combined with biochemical and functional analyses, we identify molecular defects linked to aging and AD. Our findings suggest that aging and AD create a cell-autonomous vulnerable state that leads to spontaneous lysosomal damage, delayed repair of compromised lysosomes, intra-neuronal deposition of Aβ42 and tau and secretion of inflammatory cytokines in human tNeurons without need of external seeding or stressors. These findings illuminate a fundamental aspect of disease mechanisms that may lead to novel therapeutic insights.

## Comparing proteostasis deficits and epigenetic signatures in fibroblasts and tNeurons from aged and AD donors

We harnessed a previously established paradigm to transdifferentiate human fibroblasts into cortical neurons using transcription factors *Brn2*, *Ascl1*, *Myt1l*, and *Ngn2* (herein BAMN factors) (Fig.1a). Human fibroblasts were collected from 8 healthy young (age: 25.6 ± 4.9) and 12 aged donors (age: 70.3 ± 5.9), as well as 16 aged patients with sAD (herein aged/sAD, age: 70.4 ± 9.2). As described below, fibroblasts from 5 middle-aged fAD patients carrying *PSEN1* mutations (herein fAD-PSEN1, age: 47.2 ± 10.2) were subsequently derived for validation experiments (Extended Data Table 1). Analysis of epigenetic age- and disease-associated markers in fibroblasts indicated an expected correlation with donor’s age and disease status (Fig.1b,c), similar to those previously described in AD brain tissue^23–25^. Compared with fibroblasts of young donors, fibroblasts from aged and aged/sAD donors showed an increase in DNA damage measured by γ-H2AX puncta (Fig.1b) and a global loss of epigenetic marker, histone 3 lysine 9 trimethylation (H3K9me3) (Fig.1c). Of note, we observed Histone H4 lysine 16 acetylation (H4K16ac) enriched during aging but reduced with AD (Fig.1c), which recapitulates findings in a genome-wide assessment of histone modifications in brain during normal aging and AD^25^. We next assessed if human fibroblasts exhibit aging- and AD-linked proteostasis defects by monitoring the accumulation of ubiquitin-positive (Ub+) puncta and formation of autophagic structures marked by receptor p62/SQSTM1. Interestingly, we did not observe Ub+ or p62/SQSTM1 inclusions in fibroblasts under basal conditions (Extended Data Fig.1a,b). However, when fibroblasts were exposed to proteotoxic stress by treatment with either proteasome inhibitor Bortezomib (BTZ) or lysosomal inhibitor Chloroquine (CQ), we observed dramatic differences in proteostasis impairment between young, aged and aged/sAD donors derived fibroblasts. Compared to fibroblasts from young donors, there was a moderate increase in the accumulation of Ub+ and p62/SQSTM1 puncta in aged fibroblasts and a much higher increase in puncta in aged/sAD fibroblasts (Extended Data Fig.1a,b). These experiments indicate an age-dependent increase in proteostasis vulnerability to proteotoxic stress that is exacerbated in aged/sAD fibroblasts compared to healthy aged fibroblasts.

We next implemented a combined transcription-factor and small-molecule protocol to generate tNeurons with cortical neuron characteristics at post-induction day (PID) 35 to 42 (Fig.1d, and Extended Data Fig.2a,b). Overall, our protocol could efficiently transdifferentiate fibroblasts from aged and aged/sAD donors with only a slight reduction in fibroblast-to-neuron conversion efficiency compared to those from young donors, as judged by several neuronal markers, including Tuj1, MAP2 and NeuN as well as synaptic development monitored by Syn-1 (Extended Data Fig.2c,d). Neuronal transdifferentiation retained epigenetic hallmarks of aging and disease observed in the donor fibroblasts, including aged- and aged/sAD-related increases in DNA damage (Fig.1e), global loss of H3K9me3 and H4K16ac redistribution (Fig.1f). Thus, direct conversion of donor fibroblasts to tNeurons afforded a system to study human neuronal aging and disease. We next used the formation of Ub+ and p62/SQSTM1 inclusions to examine if tNeurons also exhibited aging- and AD-linked proteostasis deficits. Surprisingly, tNeurons now displayed aging- and sAD-linked proteostasis deficits even in the absence of any proteostasis stressor. We observed Ub+ and p62/SQSTM1 puncta in aged tNeurons that were increased in aged/sAD tNeurons under basal unstressed conditions (Fig.1g, and Extended Data Fig.3a). No such inclusions were observed in tNeurons derived from young donors, indicating that the proteostasis dysfunction does not originate from the conversion protocol. Notably, the originating fibroblasts did not contain Ub+ and p62/SQSTM1 puncta under basal conditions, but exhibited ageing and AD-linked proteostasis deficits only upon BTZ or CQ treatment (Extended Data Fig.1). These experiments indicate that donor fibroblasts and the derived neurons retain the epigenetic marks and proteostasis impairments associated with aging and sAD, but also indicate that proteostasis deficits present in fibroblasts are exacerbated in tNeurons, resulting in a basal level of misfolded protein accumulation.

## Spontaneous formation of proteotoxic inclusions in tNeurons from aged and aged/sAD donors

Since aging and AD are characterized by the accumulation of misfolded toxic deposits, we examined the presence of AD-associated protein pathologies in patient-derived tNeurons. We monitored the presence of intra-cellular total Aβ, as well as its toxic isoform Aβ42, and the accumulation of phosphorylated Tau (pTau) (Fig.1g, and Extended Data Fig.3a). None of these proteotoxic pathologies were observed in tNeurons derived from young donors, but they were observed in those from aged donors. Strikingly, there was a dramatic increase in all these protein pathologies in tNeurons derived from aged/sAD donors. A sensitive ELISA detection assay confirmed an increase in Aβ42 levels in lysates of aged/sAD tNeurons (Extended Data Fig.3b). The clear progressive enrichment in these AD phenotypes between aged and aged/sAD tNeurons suggests a cooperative contribution of aging to increased Aβ42 and pTau neuronal deposition, which are clear hallmarks of AD. We also observed increased TDP-43 pathology in aged and aged/sAD tNeurons (Fig.1g, and Extended Data Fig.3a), including cytoplasmic staining of the normally nuclear TDP-43 and hyper-phosphorylated TDP-43 (pTDP-43). TDP-43 pathology is a hallmark of amyotrophic lateral sclerosis (ALS) and frontotemporal dementia (FTD)^26^, but occurs in 23–50% of AD cases^27,28^. Importantly, these deposits were observed under basal conditions, without seeding or stressors, indicating they form spontaneously in these cells. Of note, pTau inclusions partially colocalized with p62/SQSTM1 puncta while pTDP-43 partially colocalized with Ub+ puncta (Extended Data Fig.3c), linking cell-intrinsic deficits in neuronal proteostasis to AD-related protein pathologies. These findings indicate tNeurons exhibit a more limited proteostasis capacity than their parental fibroblasts. Such vulnerability is exacerbated by aging and AD deficits to cooperatively promote the cell-intrinsic accumulation of multiple AD-related proteins in human neurons.

## Quantitative proteomics uncovers how aging and AD alter human neuronal proteome

While the transcriptome of AD patient-derived neurons has been extensively characterized^29–31^, how aging and AD affect the proteome of patient-derived neurons has not been examined. We carried out quantitative proteomic analyses of young, aged and aged/sAD tNeurons at PID 40. TMT-proteomics revealed a total of 6,015 proteins with more than two unique peptides, allowing us to identify the top proteomic hits affected by aging and sAD (Extended Data Fig.4a,b). Combining GO and integrated network analysis revealed that the common top-ranked pathways altered by aging and AD involve proteostasis and organelle homeostasis, including endosome-lysosomal function, chaperones, mitochondrial function, lipid signaling as well as synapses, actin cytoskeleton and inflammation (Fig.2a, and Extended Data Fig.4c). These included age-related up-regulation of some proteins, e.g. modulating synapse (e.g. RAB27B and CADPS) and mitochondria (e.g. HK2), while proteins in the endosome-lysosomal pathway (e.g. CLU, CD63, CTSC and TMEM175) and chaperones (e.g. SACS) were mostly down-regulated with aging Comparing proteomic changes between aged and young tNeurons and between aged/sAD and young tNeurons (Extended Data Fig.S4d,e), revealed a core set of proteomic changes that overlapped between aged and aged/sAD tNeurons. These changes included proteins with function in the endosome-lysosomal, apoptosis, cytoskeleton and ER pathways. Over 94% of overlapped proteins show the same direction of expression changes in both aged and aged/sAD tNeurons. We also observed sAD-specific changes when comparing aged/sAD and aged tNeurons, including up-regulation of proteins in the endosome-lysosome (e.g. CLU and TMEM175), mitochondria (e.g. CHDH), inflammation (e.g. PYCARD and CRLF1) and synapse (e.g. SORCS2) and down-regulation of membrane and vesicular trafficking proteins in sAD (Fig.2a).

Cluster analysis of our proteomic data based on the similarity of protein expression across young, aged and aged/sAD tNeurons identified related subsets of proteins with unique trajectories of change during aging and AD (Fig.2c). We identified 13 major categories corresponding to specific biological processes with distinct changes during normal aging and AD. For instance, clusters E and G exhibited increased protein expression from young to aged to aged/sAD tNeurons. These included proteins involved in aggresome formation, ER, mitochondrial translation and apoptosis. In contrast, clusters H and M exhibited decreased protein expression from young to aged to aged/sAD tNeurons. These clusters included proteins regulating lysosome, lipid metabolism and axon maintenance. These results reveal that aging and AD broadly impact the neuronal proteome of different cellular pathways, likely contributing to shared aging and AD deficits. Remarkably, many of the proteome changes in aged and aged/sAD tNeurons correspond to proteins encoded by risk genes in neurodegenerative diseases (Fig.2b), most notably those involving the endosome-lysosomal system. Indeed, the endosome-lysosomal system appears to be a major pathway affected by aging and AD in tNeurons, with many proteins associated with lysosomes (e.g. ACE, ANPEP, CLU, SLC15A3) or lysosomal quality control (LQC) altered in aging (e.g. CNN2, HspB1) and AD (e.g. DPP7, PLBD2, PLD3, TAGLN). Our quantitative proteomics study reveals a comprehensive landscape of the proteomic features of neurons from aged and aged/sAD donors. These analyses suggest that proteome changes in aged neurons impair specific cellular pathways, rendering them vulnerable to further insults, such as stress events or risk gene mutations, to bring about age-related neurodegenerative diseases.

## tNeurons derived from fAD donors carrying *PSEN1* mutations exhibit protein pathologies and proteome alterations

Since tNeurons derived from aged/sAD donors exhibit proteostasis deficits and amyloid deposits characteristic of AD, we next examined tNeurons derived from fibroblasts of fAD donors carrying *PSEN1* mutations (herein fAD-PSEN1). *PSEN1* mutants affect APP processing and elicit early development of AD through increased production of Aβ42^32^. These tNeurons exhibited proteostasis deficits, manifested as basal accumulation of Ub+ and p62/SQSTM1 puncta. Additionally, fAD-PSEN1 tNeurons also contained Aβ, pTau and p-TDP43 protein pathologies in even higher levels than aged/sAD tNeurons (Extended Data Fig.5a,b).

We next analyzed the neuronal proteome of fAD-PSEN1 tNeurons using quantitative proteomics. We identified top proteomic hits and common top-ranked pathways associated with fAD-PSEN1 as compared with aged/sAD tNeurons (Extended Data Fig.5c-e) or young tNeurons (Extended Data Fig.5f-h). Consistent with the younger age of the donors (47.2 y/o for fAD-PSEN1 vs. 70.4 y/o for aged/sAD), fAD-PSEN1 tNeurons had reduced expression of proteins in the “Aging” category (Extended Data Fig.5d). The most dramatic change in fAD-PSEN1 tNeurons was the down-regulation of “mitochondrial proteins”, including mitoribosomes, as well as “lipid” and “neuron projection” categories. The molecular proteome signatures of *PSEN1* mutations in neurons reveal common changes in genetic and sporadic form of AD and aging-associated phenotypes. These analyses indicate that tNeurons can reveal the impact of pathogenic mutations in AD on neuronal proteostasis.

## Organelle morphology changes and constitutive lysosomal damage in aged human tNeurons are exacerbated by AD

The centrality of endosome-lysosomal proteome changes in aged and AD tNeurons led us to examine their endosome-lysosomal integrity and function. Transmission electron microscopy (TEM) characterized how aging and sAD affect lysosomal ultrastructure in human tNeurons. There was a progressive increase in the size of individual lysosomes within the cell body and proximal neurites between young, aged and aged/sAD tNeurons as well as an aging- and AD-dependent presence of electron dense deposits in lysosomes (Fig.3a). We observed small electron-dense granules proximal to the lysosomal membrane in aged tNeurons and large electron-dense granules in aged/sAD tNeurons. TEM also showed regularly shaped mitochondria in young tNeurons, whereas in aged and even more in aged/sAD tNeurons mitochondria were relocalized to closely surround the enlarged lysosomes within the cell body, suggesting increased mitochondria-lysosome contacts in AD neurons (Fig.3a).

Based on our proteomic and imaging analyses, we hypothesized that the cellular state of aged and AD tNeurons affects lysosomal resilience to damage and/or restoration from damage (Fig.3b). Damaged lysosomes are recognized by a complex LQC machinery to facilitate either repair or clearance, to protect cells from death caused by lysosomal membrane permeabilization and leakage^33–36^. Two well-characterized LQC mechanisms involve ESCRT proteins (ESCRTs) and lectins called Galectins (Extended Data Fig.6a). Mildly damaged lysosomes recruit ESCRTs to repair small membrane wounds. More severely damaged lysosomes recruit Galectins to bind to luminal glycosylated proteins and promote lysosomal clearance by autophagy (i.e. lysophagy).

We used quantitative immunostaining assays measuring recruitment of ESCRTs and Galectins to lysosomes to assess lysosomal integrity under basal unstressed conditions. In tNeurons derived from young donors, we did not detect appreciable levels of damaged lysosomes. In contrast, we observed a slight increase in lysosomal damage markers in tNeurons derived from aged donors. Strikingly, the number and intensity of CHMP2B- and Galectin-3-containing puncta in lysosomes of aged/sAD tNeurons was significantly higher compared with aged tNeurons, suggesting that lysosomal integrity is more selectively affected in sAD in basal conditions, without added stressors (Fig.3c, and Extended Data Fig.6b,c). Nonetheless, monitoring activated Caspase-3/7 levels showed no spontaneous apoptotic death under baseline conditions in all tNeuron groups at PID 35 to 42 (Extended Data Fig.6d). Together, these analyses imply that aged/sAD tNeurons have constitutively fragile lysosomes that recruit the LQC machinery to prevent cell death. Interestingly, some aged/sAD tNeurons showed aberrant accumulations of CHMP2B adjacent to the plasma membrane and neurite branch points (Extended Data Fig.6b), consistent with previous reports that CHMP2B participates in repair of both plasma and lysosomal membrane damage^33,34,37^. We conclude that lysosomes in tNeurons from aged/sAD patients, but not those from young and aged donors, carry a significant burden of constitutive lysosomal damage.

We also examined lysosomal integrity in the donor fibroblasts. Notably, we did not observe either CHMP2B-positive and Galectin-3-positive puncta in any of the human fibroblasts from any donor groups under basal conditions, in contrast to what we observe in aged and aged/sAD tNeurons (Fig.3d). Thus, transdifferentiation of fibroblasts into tNeurons enhances the aging and AD dependent lysosomal vulnerability, leading to constitutive lysosomal damage. This supports the idea that aging synergizes with sAD to increase neuronal lysosomal damage and homeostasis deficits. It is interesting that lysosomal damage is observed here in aged and AD tNeurons constitutively, in the absence of exogenous seeding or stress, unlike previous studies where uptake of extra-cellular amyloids from Aβ or tau leads to lysosomal damage and neuronal cell death^11–14,38^. While accumulation of exogenous amyloids may indeed contribute to lysosomal damage, perhaps in later stages of AD disease, our data indicates aging and sAD cause cell-autonomous, intrinsic vulnerabilities leading to constitutive lysosomal damage in human neurons.

## Aging and sAD impact neuronal lysosomal repair pathways

To test if the constitutive lysosomal damage results from impaired LQC capacity in aged and aged/sAD neurons, we examined the repair dynamics in different tNeuron groups. A short incubation with a well-validated widely used lysosomotropic tool compound, L-leucyl-L-leucine O-methyl ester (LLOME)^39–41^, was followed by a chase without LLOME to assess repair kinetics. To account for the presence of baseline lysosomal damage marked by ESCRT-III CHMP2B puncta, we chose to monitor the spatiotemporal change of lysosomal damage by following ESCRT-0 HGS because its baseline distribution was lower and comparable across all tNeuron groups (Fig.3e). Lysosomal damage with a 30-min LLOME treatment caused an increase in HGS puncta number for all tNeuron groups. Compared with young tNeurons, the number of HGS puncta moderately increased in aged tNeurons and substantially increased in aged/sAD tNeurons. We then monitored the kinetics of decrease in HGS puncta following LLOME washout. The half-life estimates (t_1/2_) of HGS puncta decrease revealed differences in the efficiency of lysosomal repair after LLOME washout. In young tNeurons, HGS puncta rapidly returned to baseline levels after 2 hr. In contrast, aged tNeurons exhibited a slight delay in the dissipation of HGS puncta while aged/sAD tNeurons exhibited a 3-fold delay returning to baseline levels (Fig.3e).

## Impact of lysosomal damage on other proteostasis pathways

Lysosomal damage and repair have been linked to several proteostasis pathways^42,43^. A previous study reported that cytosolic chaperone Hsp70 binds to and stabilizes damaged lysosomes^43,44^. Additionally, RNA-containing stress granules are reported to plug holes in the lysosomal membrane^36^. We thus examined whether aging, AD and lysosomal damage affect the cellular distribution of cytoplasmic Hsp70 and nuclear RNA-binding protein TDP-43 in human tNeurons (Extended Data Fig.6e). We observed increasing levels of cytoplasmic TDP-43 in both aged and even more in aged/sAD tNeurons under basal conditions (Fig.1g and Extended Data Fig.6e), as well as an increase in TDP43 mislocalization to lysosomes, particularly in LLOME-treated aged/sAD tNeurons (Extended Data Fig.6e). Hsp70 localization was also affected. While mostly diffuse in tNeurons under basal conditions or in LLOME-treated tNeurons from young donors, Hsp70 was strongly recruited to LLOME-mediated damaged lysosomes in aged tNeurons and exacerbated in aged/sAD tNeurons. These experiments highlight the interdependence of lysosomal deficits with alterations in additional cellular pathways.

Because aged and aged/sAD tNeurons altered the mitochondrial proteome (Fig.2a) and exhibited increased mitochondria-lysosome contacts (Fig.3a), we considered a potential interplay between lysosomal damage and mitochondrial dysfunction in patient neurons. We used tetramethylrhodamine, ethyl ester (TMRE) which accumulates only in metabolically active mitochondria. Indeed, TMRE fluorescence was reduced in all tNeurons treated with an uncoupler of the mitochondrial respiratory chain, carbonyl cyanide 4-(trifluoromethoxy)phenylhydrazone (FCCP). We then measured mitochondrial membrane potentials in tNeurons under basal conditions and following a 30-min LLOME treatment to damage lysosomes (Fig.3f). Even under basal conditions, TMRE fluorescence was significantly lower in aged and aged/sAD tNeurons compared to young tNeurons, indicative of aged- and AD-reduced mitochondrial function (Fig.3f). Interestingly, lysosomal damage with LLOME led to decreased TMRE fluorescence intensity in young and aged tNeurons. Even in aged/sAD tNeurons, the low mitochondrial membrane potential was further reduced by LLOME treatment. These experiments indicate that lysosomal damage impairs mitochondrial function, with aged and aged/sAD tNeurons being particularly vulnerable to impairment.

We next asked if lysosomal damage affects lysosomal acidification in tNeurons, essential for lysosomal function. Compared to aged tNeurons, aged/sAD tNeurons exhibited a slight loss of lysosomal acidification at baseline, which was exacerbated in the presence of LLOME (Extended Data Fig.6f). Since previous findings in mouse models at early AD stages showed that Aβ preferentially accumulates in poorly acidified lysosomes^45^, we next wondered if Aβ42 deposits localize to lysosomes in AD patient neurons. Indeed, Aβ42 colocalized with both LAMP1 (Fig.3g) and LC3B (Extended Data Fig.6g) in aged/sAD tNeurons, indicating a link between lysosomal damage and amyloid accumulation.

## Lysosomal and mitochondrial phenotypes in tNeurons from fAD-PSEN1 donors

We next investigated lysosomal damage and dysfunction in fAD-PSEN1 compared to aged/sAD tNeurons. Under basal conditions, CHMP2B- and Galecin-3-containing puncta were exclusively increased in aged/sAD tNeurons but not in fAD-PSEN1 tNeurons (Extended Data Fig.7a). Thus, aging is a more potent factor inducing constitutive lysosomal damage in AD tNeurons than *PSEN1* mutations in the absence of stressors. We next examined lysosomal repair kinetics through monitoring the spatiotemporal change of HGS puncta accumulation during an LLOME pulse and their dissipation following LLOME washout. Both aged/sAD and fAD-PSEN1 tNeurons formed significantly higher number of HGS puncta that young tNeurons during LLOME treatment and exhibited slower kinetics of HGS disappearance following washout. Relative to young tNeurons, the half-life estimate (t_1/2_) of HGS puncta dissipation increased by ~2-fold in fAD-PSEN1 and ~3-fold in aged/sAD (Extended Data Fig.7b). Analysis of mitochondrial membrane potential and lysosomal acidification also revealed phenotypic similarities between fAD-PSEN1 and aged/sAD tNeurons (Extended Data Fig.7c,d). These results indicate that fAD-PSEN1 tNeurons exhibit lower levels of lysosomal damage under basal conditions but, as observed in aged/sAD tNeurons, have increased Aβ burden and deficits in lysosomal repair and organellar function.

## Comparing lysosomal damage markers with Aβ burden in aged and AD tNeurons

Since endocytosis of amyloid fibrils is proposed to cause lysosomal damage^11,12^, we next considered whether lysosomal damage in aged and AD tNeurons is a consequence of insults caused by Aβ42. To this end, we analyzed the correlation between intra-cellular Aβ42 levels and lysosomal damage in young, aged and aged/sAD tNeurons (Fig.3h and Extended Data Fig.8a). Total levels of Aβ42 were quantified as in Extended Data Fig.3a and 5a and lysosomal damage was measured by the number of Galectin-3 and CHMP2B puncta as in Fig.3c and Extended Data Fig.7a. When comparing between young, aged and aged/sAD tNeurons, we observed a moderate positive correlation (R^2^ = 0.52), consistent with an aging- and sAD-dependent association between Aβ42 and Galectin-3 puncta number (Fig.3h). Similar results were observed when comparing Aβ42 and CHMP2B puncta number (Extended Data Fig.8a), or when comparing total intra-cellular Aβ levels and lysosomal damage markers (Extended Data Fig.8b). We next included fAD-PSEN1 tNeurons, which exhibit high levels of intra-cellular Aβ42. Interestingly, this led to a negligible correlation between Aβ burden and lysosomal damage, measured either via Galectin-3 (R^2^ = 0.16) or CHMP2B (R^2^ = 0.09) at basal conditions (Fig.3h and Extended Data Fig.8a). Similar results were also observed when comparing total intra-cellular Aβ levels and lysosomal damage markers in fAD-PSEN1 tNeurons (Extended Data Fig.8b). These experiments suggests that lysosomal dysfunction in the aged/sAD tNeurons promotes Aβ42 accumulation rather that amyloid presence leading to constitutive lysosomal damage.

## Elevation of lysosomal damage markers associates with amyloid pathology in brain tissue of AD mouse models and patients

To link our *in vitro* findings in aged and AD tNeurons to pathophysiological conditions *in vivo*, we carried out histopathological analyses of post-mortem cerebral cortex of both AD patients and mouse models of aging and AD (Fig.4a). To examine whether brain aging is characterized by aberrant lysosomal damage signatures in neurons, we immunostained the neocortex of young (3 month) and aged (20 to 24 month) mice for Galectin-3 and LAMP1 (Extended Data Fig.9a,b). In aged mice, we observed a moderate increase in Galectin-3 immunoreactivity colocalized with enlarged LAMP1-positive lysosomes in neurons, visualized by MAP2 staining (Extended Data Fig.9b). This supports the idea that aging-linked lysosomal damage in neurons is a vulnerability factor contributing to neurodegeneration.

We next examined the link between lysosomal and proteostasis pathologies in brain sections from a mouse model for AD expressing human APP carrying the Swedish (K670N/M671L) and London (V717I) mutations (APP^Lon/Swe^)^46^ (Extended Data Fig.10a). Neurons in the neocortex were visualized by MAP2 staining, and lysosomes by LAMP1. Similar to our aged/AD tNeurons, we observed a widespread accumulation of CHMP2B-positive and LAMP1-positive lysosomes inside neurons in AD mice but not the non-transgenic (NTg) controls (Fig.4b, and Extended Data Fig.10b). We also observed LAMP1-positive clumps that colocalized with lysosomal damage markers CHMP2B (Fig.4b, and Extended Data Fig.10b), Galectin-3 (Extended Data Fig.10c) and chaperone Hsp70 (Extended Data Fig.10d). These clumps and damaged lysosomal markers were observed both in the perinuclear region within individual pyramidal neurons, as well as in the extra-cellular space. The analysis of Aβ42-containing amyloid plaques also reflected our results in AD tNeuron models, since Aβ42 inclusions colocalized with lysosomal structures. Pyramidal neurons of APP^Lon/Swe^ mice proximal to Aβ plaques contained intra-neuronal Aβ42 deposits within lysosomes (Fig.4b). Unlike tNeurons, we also observed extra-cellular Aβ deposits in the brain sections, which were associated with large protein clumps immuno-positive for CHMP2B and Galectin-3 (Extended Data Fig.10b). These extra-cellular Aβ deposits were associated with severe brain damage regions devoid of MAP2 staining (Extended Data Fig.10b-d), suggesting that they result from neuronal death linked to lysosomal damage.

Immunostaining of human brain sections of AD patients also revealed both intra-neuronal and global elevation of Aβ, CHMP2B and Galectin-3 immunoreactivity colocalizing with LAMP2, compared with healthy control (HC) individuals (Fig.4c, and Extended Data Fig.11a-d). Of note, Galectin-3 is strongly associated with amyloid plaques^47,48^. Interestingly, we identified two distinct patterns of CHMP2B staining in AD patient brains (Fig.4c, and Extended Data Fig.11a). In the perinuclear region of neurons of AD brain sections, CHMP2B primarily colocalized with LAMP2-positive lysosomes. We also found thin, thread-like, CHMP2B-positive structures of variable length devoid of LAMP2 positivity, in AD cerebral cortexes. Also consistent with our tNeuron findings, we observed increased colocalization of Galectin-3 with LAMP2 in neurons of AD patients (Extended Data Fig.11b) and Aβ deposits associated with LAMP2-positive lysosomes in both extra-cellular and intra-neuronal plaques using 6E10 antibody in AD patient’s cortex (Fig.4c, and Extended Data Fig.11c). Thus, despite their increased complexity, brains of human AD patients and mouse models of aging and AD all reveal intra-neuronal and global increase in lysosomal damage markers accompanying co-aggregation with amyloid plaques and neuritic degeneration, consistent with the phenotypes of our tNeuron models.

## Lysosomal damage potentiates cell-autonomous inflammatory activation in AD tNeurons

The proteomic analyses revealed that aged and aged/sAD tNeurons upregulates a protein network involved in cytokine signaling, including inflammasome adaptor protein PYCARD/ASC (Fig.5a). The inflammasome protein complex consisting of NLRP3 (sensor), PYCARD/ASC (adaptor) and protease caspase-1 (effector). While activation of NLRP3 inflammasomes in microglia has been linked to AD pathogenesis^49^, this result suggested this innate immunity inflammatory response is also up-regulated in AD neurons. We asked if lysosomal damage contributes to inflammasome activation leading to cell-autonomous cytokine secretion from human tNeurons (Fig.5b). Immunostaining for NLRP3 and PYCARD/ASC showed that under basal conditions aged and fAD-PSEN1 tNeurons contained 2.7% inflammasome-positive neurons while aged/sAD tNeurons contained 4.6% inflammasome-positive neurons per field of view (Fig.5c). Notably, a 3-hr LLOME treatment led to widespread activation of NLRP3 inflammasomes in aged and AD tNeurons, but less so in young tNeurons. Following lysosomal damage stress, the incidence of inflammasome-positive neurons was increased by 35–40% in aged/sAD tNeurons compared with 18–30% in aged and fAD-PSEN1 donors. Thus, aged and AD tNeurons possess a relatively low-level inflammasome activation at baseline, but their higher vulnerability to lysosomal damage leads to a potent activation of inflammasomes.

We examined whether tNeurons from our different donor groups themselves secrete inflammatory factors under basal conditions by measuring a panel of inflammatory factors in the conditioned medium from untreated tNeurons. Multiplex cytokine profiling using Luminex assays showed an age- and AD-dependent increase in levels of secreted pro-inflammatory cytokines (e.g. IL-6 and IFN-γ) compared to young; interestingly, aged/sAD tNeurons also induced anti-inflammatory cytokines (e.g. IL-1β, IL-4 and IL-15) and chemokines (e.g. CCL2 and CXCL12) (Fig.5d, and Extended Data Fig.12a). We next tested the hypothesis that inflammatory factor secretion from tNeurons is enhanced by lysosomal damage. As shown above, tNeurons of young donors do not mount an appreciable basal inflammatory response. We subjected tNeurons of young donors to a chronic and low-dose treatment with LLOME (0.1 mM for 7 days) to elicit a sublethal lysosomal damage state. Notably, this treatment led to increased secretion of pro-inflammatory cytokines (e.g. IL-1β, IL-6 and IFN-γ) and chemokines (e.g. CCL2) from young tNeurons (Fig.5e, and Extended Data Fig.12a). This indicates that lysosomal damage can promote cell-autonomous secretion of inflammatory factors in human neurons. Pearson’s correlation analysis identified a moderate and positive correlation between total cytokine levels in the conditioned medium of aged and aged/sAD tNeurons, as well as between young tNeurons treated with LLOME and AD tNeurons (Fig.5f).

To further link lysosomal damage to neuronal inflammatory activation, we asked if a previously identified small molecule, C381, that ameliorates lysosomal dysfunction can reduce secretion of inflammatory factors in aged and AD tNeurons. C381 promotes lysosomal acidification by targeting v-ATPase complexes and improves resilience to damage in cellular and mouse models of neurodegenerative diseases^50^. We treated aged/sAD and fAD-PSEN1tNeurons with and without C381 (3.1 μM for 7 days) and measured cytokine secretion as above. Strikingly, amelioration of lysosomal damage by C381 pre-treatment significantly reduced the levels of secreted pro-inflammatory cytokines (e.g. IL-6 and IL-15) and chemokines (e.g. CCL2 and CXCL12) in both aged/sAD and fAD-PSEN1 tNeurons (Fig.5g, and Extended Data Fig.12b).

To relate these cell-based findings of tNeuron cytokine secretion relate to neuron immune activation in a physiological disease-relevant context we examined cytokine expression in existing human datasets of single-nucleus transcriptomic analysis of neurons, astrocytes, microglia and oligodendrocytes from post-mortem cortex from HC donors. As expected, given their immune-active function, microglia have high transcript levels of cytokines, chemokines and other inflammatory molecules (Extended Data Fig.13). However, disease linked neurons and glial cells also contained cytokine and chemokine transcripts, albeit at lower levels^29^. Because CSF cytokines have been increasingly recognized as potential biomarkers for AD^51^, we next conducted an unbiased analysis of CSF proteins in 50 HC and 29 AD donors in a search for biomarkers. We found higher CSF levels of IL-15 and IL-20, and lower CSF levels of IL-1A, IL-1RA, VEGFA and TNFRSF6B in AD than in HC (Extended Data Fig.14 and Table 3). Of note, IL-15 found in both tNeuron conditioned medium and CSF of AD patients (Fig.5d, Extended Data Fig.12 and Fig.14), has been positively correlated with age of onset in AD^52^. In intact brains, the cytokine secretion of microglia likely dominates the inflammatory response in AD as expected. However, we propose the neuronal secretion of a subset of cytokines caused by cell-intrinsic lysosomal dysfunction during aging and AD may initiate an inflammatory cascade that propagates to other brain cell types. These experiments suggest ameliorating lysosomal function may elicit neuroprotective effects by inhibiting inflammatory factor secretion in AD patient neurons.

## Rescuing lysosomal function ameliorates multiple AD pathologies in tNeurons

We extended the above small molecule approach to investigate the link between lysosomal impairment, neuronal cell death and Aβ deposits in tNeurons (Fig.6a). Supporting a lysosome protective function, pre-incubation of aged and AD tNeurons with C381 (3.1 μM for 7 days) reduced the number of detectable CHMP2B and Galectin-3 puncta on lysosomes in aged, aged/sAD and fAD-PSEN1 tNeurons under basal conditions (Fig.6b). We thus tested if C381 can help restore lysosomal function and protect from neuronal death following LLOME damage. Since lysosomal hydrolases, such as Cathepsins, are optimally active at pH 4–5, the observed lysosomal deacidification caused by either aging, AD and LLOME (Extended Data Fig.6f and 7d) should abrogate their activity. Indeed, Cathepsin-B hydrolysis of its Magic Red substrate was reduced in aged and aged/sAD tNeurons compared with young tNeurons under basal conditions and was dramatically lost upon treatment with LLOME (Fig.6c). However, C381 treatment prevented the loss of Cathepsin-B activity upon LLOME-mediated damage and restored Cathepsin-B activity to near-normal levels. Aged/sAD and fAD-PSEN1 tNeurons were exquisitely sensitive to LLOME-mediated cell death, as measured by Caspase-3/7 activation (Fig.6d). Notably, C381 was strongly neuroprotective from LLOME-mediated cell apoptotic death in AD tNeurons, reducing Caspase-3/7 activation in both aged/sAD and fAD-PSEN1 tNeurons by ~51%.

We next tested whether lysosomal dysfunction in aged and AD tNeurons contributes to cell-autonomous Aβ42 deposits. To this end, we used small molecules that either ameliorate or further impair lysosomal homeostasis, and measured Aβ42 deposits in aged, aged/AD and fAD-PSEN1 tNeurons (Fig.6e,f). To ameliorate lysosomal function, we used two molecules in addition to C381 (Fig.6e). Thioperamide rescues lysosomal lipid metabolism deficits and lysosomal damage by increasing lysosomal phospholipid, bis(monoacylglycero)phosphate (BMP)^53^. NCT-504 upregulates ESCRT transcripts and promotes ESCRT-mediated degradation^54^. To impair lysosomal function, we used LLOME, which induces lysosome damage or calcium chelator BAPTA-AM, which inhibits lysosomal repair^34^. We incubated tNeurons with lysosome-targeting compounds for 2 days and performed immunofluorescence analysis of Aβ42 deposits (Fig.6f). Increasing lysosomal damage by either LLOME or BAPTA-AM significantly increased intra-neuronal Aβ42 levels in aged/sAD and fAD-PSEN1 tNeurons. Strikingly, incubating tNeurons with any of the compounds ameliorating lysosomal function, C381, thioperamide or NCT-504, significantly reduced the levels of Aβ42 deposits in aged and AD neurons (Fig.6f; aged tNeurons by ~21–36%; aged/sAD tNeurons by ~28–38%; fAD-PSEN1 tNeurons by ~20–46%). These experiment support the connection between neuronal lysosomal dysfunction and the cell-intrinsic generation of intra-neuronal Aβ42 deposits.

## Discussion

Here we demonstrate that neuronal transdifferentiation provides a powerful approach to study cellular and mechanistic aspects of human brain aging and neurodegenerative disease caused by stochastic AD events. While human iPS cells have great potential to model genetic disorders^55^ by rejuvenating the transcriptome and proteome their erase access to the contribution of aging to neurodegenerative diseases^16,17,56,57^. Building on previous studies showing that tNeurons derived from human dermal fibroblasts preserve hallmarks of aging, including epigenetic, transcriptomic and mitochondrial signatures^17,57–59^, we used human tNeurons to reveal proteomic signature of aging and AD and identify proteostasis pathways selectively affected by aging and AD. Notably, the patient-derived neurons exhibited lower proteostasis capacity than their fibroblasts of origin, which causes the higher vulnerability of the brain to AD during aging. Our work further suggests that tNeurons may be used to evaluate small molecules possessing neuroprotective effects against aging and AD pathologies.

The proteostasis and organelle homeostasis deficits we observed in human aged and AD tNeurons are accompanied by characteristic AD-related protein pathologies, including Aβ, pTau and TDP-43 deposits (Fig.1g, Extended Data Fig.3 and Extended Data Fig.5a,b) as well as inflammasome activation and cytokine secretion (Fig.5c,d and Extended Data Fig.12a). Our results indicate these AD phenotypes arise cell-autonomously from the intrinsic dysregulation of proteostasis and lysosomal homeostasis in aged and AD neurons. Thus, these protein deposits are formed without seeding or overexpression of human disease proteins as commonly used in current model systems^60,61^. While we find compounds that ameliorate lysosomal dysfunction to improve AD phenotypes, it will be of interest to determine whether these effects are direct, or whether ameliorating lysosomal function relieved the burden of other pathways within the highly interconnected proteostasis network^5,62^.

Our tNeuron model suggests that constitutive lysosomal damage in aged and AD neurons leads to spontaneous Aβ accumulation. This conclusion is supported by several lines of evidence. First, the phenotype of lysosomal damage in tNeurons is moderately and positively correlated with aging and AD, but negligibly correlated with intra-cellular Aβ42 levels, when fAD-PSEN1 tNeurons are included (Fig.3h and Extended Data Fig.8). Additionally, we observe a reduction in spontaneously formed Aβ42 deposits when tNeurons are treated with small molecules that ameliorate lysosomal dysfunction (Fig.6). In principle, such damage may be caused by increased lysosomal vulnerability with age^63^ but may also arise from defects in repair of compromised lysosomes after damage, as observed in aged and AD tNeurons (Fig.3c–e). Of note, aging-dependent impairment of lysosomal function has also been observed along a longitudinal aging axis in other organisms^4,6,64^.

AD brains are characterized by Aβ deposits and lysosomal dysfunction^1,65^, including amyloid plaques enriched with lysosomal hydrolases, such as Cathepsin B and D^66^. However, the underlying mechanisms leading to defects linked to histopathology are poorly understood. The AD-linked phenotypes observed here in tNeurons may illuminate early neuron-specific defects linked to aging and AD, before they propagate into tissue-level damage. For instance, we find cell-intrinsic proteostasis and lysosomal deficits in aged and AD neurons leading to Aβ42 and p-Tau deposit accumulation. Neuronal death or secretion may release these toxic protein deposits that are internalized by neighboring cells, leading to further lysosomal damage of surrounding neurons and glial cells^11,12^. This process may propagate lysosomal dysfunction and Aβ aggregation, within the complex brain tissue, initiating a vicious cascade that is amplified to cause AD. Interestingly, recent reports show that intrinsically perforated endosome-lysosomes are normally present in neurons, and facilitate the seeding of cytoplasmic aggregates following internalization of preformed fibrils of a-synuclein^67^. The increased incidence of damaged lysosomes in aged and AD tNeurons would render them more vulnerable to seeding by Aβ. Similarly, cytokines secreted by increased activation of the inflammasome by damaged lysosomes may recruit and activate microglia to mount a systemic inflammatory response.

In sum, we propose tNeurons provide insights into early neuron-intrinsic cell biological processes by which loss of proteostasis and organelle homeostasis contribute to AD neuronal pathogenesis. These insights would be impossible to obtain in intact brains, where the complex interplay between cell types in the tissue establishes a vicious cycle that likely exacerbates all responses. They would also not be possible in iPSC or ESC-derived neurons, which lack the aging-linked phenotypes essential to these late-onset diseases such as AD. One corollary of our experiments is that counteracting intrinsic proteostasis and lysosomal homeostasis deficits in aged and AD tNeurons may be attractive strategies for early-stage prevention of the cascade of deleterious events in affected AD brains.

## Methods

### Experimental Model and Subject Details

#### Human subjects

De-identified human fibroblasts, post-mortem prefrontal cortex and CSF samples were acquired from individuals of various ages and disease conditions from Stanford Alzheimer’s Disease Research Center (ADRC). Cells and tissue samples were obtained under written consents from all subjects approved by Institutional Review Board of Stanford University. The cell and tissue samples collected by ADRC were not specifically for this study. For histological experiments, of these subjects, 8 were assessed as healthy control (HC) and 10 were patients with cognitive impairment (dementia due to AD). For CSF proteomics experiments, 50 were assessed as HC and 29 were patients with cognitive impairment (MCI or dementia due to AD). Age and sex demographics are detailed in Tables S1 and S3. In ADRC, all HC and individuals with cognitive impairment received neurological examinations and cognitive tests to determine cognitive status and consensus diagnosis by a team of neuropathologists. The pathological diagnosis of post-mortem tissues was made by microscopic examination of multiple brain regions using Amyloid score, Braak neurofibrillary degeneration score and CERAD neuritic plaque score.

#### HEK293T cells

The HEK293T cells are the cell line derived human embryonic kidney and were acquired directly from ATCC (CRL-1573). Cells were grown in culture medium (DMEM supplemented with GlutaMAX, 10% FBS, 1% Penicillin-Streptomycin, 1% HEPES and 1% Sodium pyruvate) (Thermo Fisher Scientific) sterilized by a 0.22 μm vacuum filter (Thermo Fisher Scientific) in a 37°C incubator with 5% CO_2_ in the air. The HEK293T cells were used for lentiviral production by transfecting lentiviral vector of interest mixed with packaging and envelope plasmid. Passaging the cells was performed with Trypsin-EDTA (Thermo Fisher Scientific) every three days.

#### Human fibroblasts

Primary human adult fibroblasts derived from clinically healthy adults and individuals diagnosed with AD were collected from shared resources in the Stanford ADRC and Coriell Institute for Medical Research, which operates the NIGMS, NIA, NINDS cell repository. Culture of primary human fibroblasts was described at https://dx.doi.org/10.17504/protocols.io.36wgq3edklk5/v1. Briefly, cells were grown in culture medium (DMEM supplemented with GlutaMAX, 10% FBS, 1% Penicillin-Streptomycin, 1% MEM NEAA, 1% Sodium pyruvate and 0.1% β-mercaptoethanol) (Thermo Fisher Scientific) sterilized by a 0.22 μm vacuum filter (Thermo Fisher Scientific) and maintained in a 37°C, 5% CO_2_ incubator. The subculture of proliferating fibroblasts used for regular experiments and neuronal transdifferentiation was typically within 3 to 7 passages. Some fibroblast lines obtained with slightly higher passage numbers were used for neuronal transdifferentiation no more than 12 passages. Passaging the cells was performed with Trypsin-EDTA every 6 to 7 days.

#### Human tNeurons

tNeurons were converted from human fibroblasts with low passage numbers using a combination of transcription factors and small molecules. This method was derived from the protocols as previously described^18,68^ and modified for this study. A detailed protocol can be found at https://dx.doi.org/10.17504/protocols.io.j8nlko6d5v5r/v1. Briefly, the lentiviral expression of Brn2, Ascl1, Myt1l and Ngn2 (also referring as BAMN factors) in human fibroblasts initiated neuronal reprogramming. Transduced cells underwent puromycin and PSA-NCAM selection and were cultured in reprogramming medium (DMEM/F12:Neurobasal (1:1) Medium, 2% B-27, 1% N-2, 0.25% GlutaMAX, 1% Penicillin-Streptomycin) (Thermo Fisher Scientific) for 15 days and then switched to maturation medium (BrainPhys Neuronal Medium (STEMCELL Technologies), 2% B-27, 1% N-2, 0.25% GlutaMAX, 1% Penicillin-Streptomycin) for additional 15 to 22 days. The culture media were supplemented with BDNF and NT-3 (neurotrophic factors; Peprotech), Doxycycline (effector for the Tet-On system; Cayman), Forskolin (cAMP activator; Sigma-Aldrich), SB 431542 (TGF-β/Activin/Nodal inhibitor; TOCRIS), Dorsomorphin (BMP inhibitor; TOCRIS) and XAV939 (Wnt inhibitor; Stemgent; removal from maturation medium). Human tNeurons resembled cortical glutamatergic neurons and were used for the evaluation of reprogramming efficiency, phenotypic characterization, and small molecule treatments after 5 weeks in culture in a 37°C, 5% CO_2_ incubator. The medium was half-changed every 2 to 4 days throughout the lifetime of the culture. More details were described in Method Details > Direct generation of neurons from human fibroblasts.

#### Mice

All mice used in this study were C57BL/6 genetic background. Mice of old age (20 to 24-month old) were obtained from the National Institute on Aging rodent colony and young age (3-month old) were obtained from Jackson Laboratories or Charles River Laboratories. All experiments used male mice. A transgenic mouse model with the expression of high levels of human APP751 carrying V717I, K670M/N671L mutations (also referring to APP^Lon/Swe^) in neurons under control of a Thy1.2 promoter has been studied in different laboratories. The APP^Lon/Swe^ mice developed amyloid plagues associated with an overproduction of Aβ42 in the neocortex and working memory deficits at 3 months old and the plaque formation spread to the hippocampus and thalamus region at 5 to 7 months old^46,69^. This study used 3 to 6-month-old APP^Lon/Swe^ and the age-matched non-transgenic mice. All mice were kept in a 12-hr light/dark cycle in a temperature- and humidity-controlled environment and provided ad libitum access to food and water. All animal care and procedures complied with the Animal Welfare Act and were in accordance with institutional guidelines and approved by the V.A. Palo Alto Committee on Animal Research and the institutional administrative panel of laboratory animal care at Stanford University.

### Lentivirus preparation

Preparation of lentiviruses was previously described at https://dx.doi.org/10.17504/protocols.io.kxygx317wg8j/v1. The FUW lentiviral vector expressing BAMN factors and EGFP is under the control of TetO promoter and M2rtTA under the control of ubiquitin promoter. The HEK293T cells (ATCC) were plated at a density of 6 × 10^6^ in a poly-L-ornithine-coated 10-cm dish and the next day co-transfected with 5 μg lentiviral transfer vector, 4 μg packaging plasmid (psPAX2) and 2.5 μg envelope plasmid (pMD2.G) using Lipofectamine 2000 in OptiMEM (Thermo Fisher Scientific). After 6-hr incubation of Lipofectamine/DNA mixture in OptiMEM, the transfection medium was replaced with fresh DMEM supplemented with GlutaMAX, 2% FBS, 1% Penicillin-Streptomycin, 1% HEPES and 0.1% BME (Thermo Fisher Scientific). The cell supernatants containing lentiviral particles were harvested after 24 hr and stored at 4°C. Cells were replenished with fresh DMEM medium with 2% FBS and cultured for additional 24 hr. The supernatants were then harvested and pooled with the first collection. To remove cell debris, the supernatants were centrifugated at 400 × G for 5 min and passed through 0.45 μm syringe filters. The clear virus-containing media can be stored at 4°C for about 1 week. For long-term storage, alternatively, the virus-containing media were spun by ultracentrifugation at 25,000 rpm for 90 min at 4°C to pellet the viruses. The viruses were resuspended in DMEM medium with 2% FBS and snap-frozen in small aliquots to store at −80°C.

### Direct generation of neurons from human fibroblasts

Human adult fibroblasts were plated at a density of 200 × 10^3^ per well of a 6-well plate coated with poly-L-ornithine. The next day, Day 0, fibroblasts were infected with lentiviruses expressing BAMN factors and M2rtTA with or without EGFP by incubating with the diluted virus-containing medium in DMEM supplemented with GlutaMAX, 2% FBS, 1% Penicillin-Streptomycin, 1% HEPES and 0.1% BME plus 4 μg/mL Polybrene for 24 hr. On Day 1, the virus-containing medium was then discarded and replaced by fresh fibroblast culture medium plus 1 μg/mL Doxycycline. On Day 2, puromycin (0.5 μg/mL) was added for selection for 48 hr. On Day 4, the transduced cells were subjected to PSA-NCAM+ selection following the manufacturer’s instructions. Briefly, 0.05% Trypsin-EDTA was added to the cells for 5 min at 37°C to dissociate them from surface and neutralized by fibroblasts culture medium, followed by the centrifugation at 300 × G for 5 min at room temperature to pellet cells. The cells were resuspended in autoMACS buffer and labeled with anti-PSA-NCAM-APC (Miltenyi Biotec) for 10 min at 4°C in the dark. After a wash by autoMACS buffer and centrifugation at 300 × G for 10 min, the cells were incubated with anti-APC MicroBeads (Miltenyi Biotec) for 15 min at 4°C in the dark. Unbound beads were then washed off and the cells were resuspended in autoMACS buffer for subsequent flow cytometry analysis and separation of magnetically PSA-NCAM-labeled and unlabeled cells. The PSA-NCAM+ cells were re-plated at a density of 50 × 10^3^ cells per cm^2^ to the plate coated with vitronectin (VTN-N, 5 μg/mL; Thermo Fisher Scientific) and laminin (rhLaminin-521, 1 μg/mL; Corning). Cells were cultured in fibroblast culture medium plus 1 μg/mL Doxycycline and the next day switched to reprogramming medium (DMEM/F12:Neurobasal (1:1) Medium, 2% B-27, 1% N-2, 0.25% GlutaMAX, 1% Penicillin-Streptomycin) (Thermo Fisher Scientific) supplemented with small molecules: 1 μg/mL Doxycycline (Cayman), 5 μM Forskolin (Sigma-Aldrich), 10 μM SB 431542 (TOCRIS), 2 μM Dorsomorphin (TOCRIS) and 2 μM XAV939 (Stemgent)). After one week, 10 ng/mL BDNF and NT-3 (Peprotech) were added to the reprogramming medium. Half of the medium was changed every 2 to 3 days. After 8 days, cells were switched to maturation medium (BrainPhys Neuronal Medium (STEMCELL Technologies), 2% B-27, 1% N-2, 0.25% GlutaMAX, 1% Penicillin-Streptomycin) supplemented with 1 μg/mL Doxycycline, 5 μM Forskolin, 10 μM SB 431542, 2 μM Dorsomorphin and 10 ng/mL BDNF and NT-3 and cultured for additional 15 to 22 days. Half of the medium was changed every 3 to 4 days. The efficiency of transdifferentiation of human fibroblasts into tNeurons was measured by the percentage of remaining transduced cells that express Tuj1, NeuN and MAP2 and the percentage of EGFP-positive cells showing neuron-like morphology.

### Quantitative proteomics (TMT, Tandem Mass Tag)

Flash frozen cell pellets were lysed in 8M urea buffer (8M urea, 150 mM NaCl, 50 mM HEPES pH 7.5, 1x EDTA-free protease inhibitor cocktail (Roche), 1x PhosSTOP phosphatase inhibitor cocktail (Roche)). Lysates were clarified by centrifugation at 17,000 × G for 15 min at 4°C. Protein concentration of the supernatant was quantified by bicinchroninic acid assay (BCA, Pierce). To reduce and alkylate cysteines, 100 μg of protein was sequentially incubated with 5 mM TCEP for 30 min, 14 mM iodoacetamide for 30 min, and 10 mM DTT for 15 min. All reactions were performed at room temperature. Next, proteins were chloroform-methanol precipitated and the pellet resuspended in 200 mM EPPS pH 8.5. Then, LysC (Wako) was added at 1:100 (LysC:protein) ratio and incubated overnight at room temperature in an orbital shaker at 1,500 rpm. The day after, samples were further digested for 5 hr at 37°C with trypsin at 1:75 (trypsin:protein) ratio in an orbital shaker at 1,500 rpm. After digestion, samples were clarified by centrifugation at 17,000 × G for 10 min. Peptide concentration of the supernatant was quantified using a quantitative colorimetric peptide assay (Thermo Fisher Scientific). For TMT labelling we used 2 different kits to label each of the TMT experiments included in this manuscript. In TMT-02, peptides from tNeuron’s samples were labelled with TMTpro-16plex tags. Same method was followed to label both TMT sets^70–72^. Briefly, 25 μg of peptides was brought to 1 μg/μL with 200 mM EPPS (pH 8.5), acetonitrile (ACN) was added to a final concentration of 30% followed by the addition of 50 μg of each TMT reagents. After 1 hr of incubation at room temperature, the reaction was stopped by the addition of 0.3% hydroxylamine (Sigma) for 15 min at room temperature. Extra information regarding both TMT sample labels is included in Table S4. After labelling, samples of each TMT were combined, desalted with tC18 SepPak solid-phase extraction cartridges (Waters), and dried in the SpeedVac. Next, desalted peptides were resuspended in 5% ACN, 10 mM NH4HCO3 pH 8. Both TMT were fractionated in a basic pH reversed phase chromatography using a HPLC equipped with a 3.5 μm Zorbax 300 Extended-C18 column (Agilent). Fractions were collected in a 96-well plate, then combined into 24 samples. Twelve of them were desalted following the C18 Stop and Go Extraction Tip (STAGE-Tip)^73^ and dried down in a SpeedVac. Finally, peptides were resuspended in 1% formic acid, 3% ACN, and analyzed by LC-MS3 in an Orbitrap Fusion Lumos (Thermo Fisher Scientific). For TMT-01 the MS was running in SPS-MS3 mode^74^. For TMT-02, instrument was equipped with FAIMS and running in RTS-MS3 mode^75–77^. More information regarding all MS parameters used for both TMT are included in Table S4. A suite of in-house pipeline (GFY-Core Version 3.8, Harvard University) was used to obtain final protein quantifications from all RAW files collected. RAW data were converted to mzXML format using a modified version of RawFileReader (5.0.7) and searched using the search engine Sequest or Comet (for TMT-01 and TM-02, respectively)^78–80^ against a mouse target-decoy protein database (downloaded from UniProt in June 2019) that included the most common contaminants. Precursor ion tolerance was set at 20 ppm and product ion tolerance at 1 Da. Cysteine carbamidomethylation (+57.0215 Da) and TMT tag (+229.1629 Da or +304.2071 Da for TMT-6plex or TMTpro-16pex, respectively) on lysine residues and peptide N-termini were set as static modifications. Up to 2 variable methionine oxidations (+15.9949 Da) and 2 miss cleavages were allowed in the searches. Peptide-spectrum matches (PSMs) were adjusted to a 1% FDR with a linear discriminant analysis^81^ and proteins were further collapsed to a final protein-level FDR of 1%. TMT quantitative values we obtained from MS3 scans. Only those with a signal-to-noise ratio > 100 and an isolation specificity > 0.7 were used for quantification. Each TMT was normalized to the total signal in each column. Quantifications included in Table S4 are represented as relative abundances. RAW files will be made available upon request. The data have been deposited in the ProteomeXchange Consortium via the PRIDE^82^ partner repository with the data set identifier PXD040834. Biological pathway and gene ontology enrichment analysis were performed using the ClueGo (Cytoscape plug-in), Enrichr or STRING.

### Caspase-3/7 activation

To detect apoptosis in tNeurons, we incubated the cells with the Caspase-3/7 substrate FAM-DEVD-FMK (ImmunoChemistry), one of the fluorochrome-labeled inhibitors of caspases that covalently and irreversibly binds to the active caspases. The green fluorescence intensity is a direct measurement of Caspase-3/7 activity. Human tNeurons were treated with DMSO or 0.5 mM LLOME for 1 hr to measure the lysosome-mediated apoptosis or pretreated with 3.1 μM C381 followed by LLOME treatment to assess the rescuing effect of C381 on lysosome-mediated apoptosis. The FAM-DEVD-FMK reagent was reconstituted in DMSO and stored at −20°C. When cells were available, the FAM-DEVD-FMK reagent was diluted with PBS 1:5 ratio and added to the cell culture medium at a dilution of 1:30 to form 1X staining solution. Cells were incubated with the FAM-DEVD-FMK solution for 30 min at 37°C. After a rinse by Apoptosis Buffer, the cells were fixed by 4% paraformaldehyde (PFA) for 15 min and counterstained with 5 μg/mL Hoechst for confocal microscope imaging.

### Fluorescence-conjugated Dextran assay for measuring endocytosis and lysosomal acidification

Fluorescein isothiocyanate (FITC)-conjugated Dextran at 40 kDa and tetramethylrhodamine (TMR)-conjugated Dextran at 10 kDa and 70 kDa (Thermo Fisher Scientific) were reconstituted in H_2_O and stored at −20°C. Human tNeurons were seeded at 5 × 10^4^ per well of a 24-well plate or 5 × 10^3^ per well of a 96-well plate. Cells were incubated with FITC-Dextran at 0.5 mg/mL for 4 hr at 37°C and rinsed by PBS, followed by a 20-hr chase in fresh culture medium to accumulate Dextran in late endosomes and lysosomes. Then, the cells were treated with DMSO or 0.25 mM LLOME for 30 min and then washed out to assess cellular endocytosis and lysosomal function recovery. The cells were fixed by 4% PFA for 15 min and prepared for imaging by the confocal microscope and CLARIOstar plate reader.

### Magic Red Cathepsin-B assay for measuring lysosomal proteolysis

Magic Red Cathepsin-B substrate, MR-(RR)2, containing arginine-arginine (RR) sequence was reconstituted in DMSO and stored at −20°C. When cells were available for an experiment, MR-(RR)2 was diluted with H_2_O 1:10 ratio and added to cell culture medium at a dilution of 1:25 to form 1X staining solution. Active Cathepsin-B cleaves MR-(RR)2 and emits fluorescence with optimal excitation of 592 nm and emission of 628 nm. To test lysosomal proteolytic capacity, human tNeurons were incubated MR-(RR)2 staining solution with for 30 min at 37°C, followed by DMSO or 0.25 mM LLOME treatment for 30 min. To evaluate the pharmacological rescuing effect, cells were pre-treated with 3.1 μM C381 for 7 days before the cells were loaded with MR-(RR)2. Cells were treated with 0.25 mM LLOME for 30 min, and then fixed by 4% PFA for 15 min for imaging by the confocal microscope and CLARIOstar plate reader.

### TMRE

To detect mitochondrial membrane potential in human tNeurons, we used the tetramethylrhodamine ethyl ester (TMRE) reagent, which accumulates in functional and polarized mitochondria according to Δψm. The TMRE reagent was reconstituted in DMSO for a stock solution at 1 mM and stored at −20°C. When tNeurons were in culture for 5 weeks, the cells were pre-treated with DMSO, 50 μM FCCP or 0.25 mM LLOME for 10 min. Then TMRE reagent was added to fresh cell culture medium at a dilution of 1:1000 along with FCCP or LLOME. Half of the old culture medium was replaced with the TMRE-containing medium in order to incubate the cells with TMRE at a final concentration of 500 nM for 30 min at 37°C. Cells were then rinsed with pre-warmed 0.2% bovine serum albumin (BSA)/PBS twice and positioned in the CLARIOstar plate reader for fluorescence measurements with setting of optimal acquisition parameters (excitation of 549 nm and emission of 575 nm).

### ELISA

To measure Aβ42 levels, human tNeurons were trypsinized, washed with ice-cold PBS and pelleted by centrifugation for 5 min at 1,000 × G. Cells were then lysed with RIPA buffer (25 mM Tris-HCl pH 7.5, 150 mM NaCl, 1% NP-40, 0.5% sodium deoxycholate) supplemented with protease inhibitors (Roche). Protein concentrations were determined by BCA assay (Thermo Fisher Scientific). For Aβ42 assay, we used human Aβ42 ELISA Kit (Thermo Fisher Scientific) to detect and quantify the levels in total tNeuron lysates. Briefly, 50 μL of the cell lysates were added to each well of a 96-well plate, followed by the incubation with Aβ42 antibody for 3 hr, anti-rabbit IgG HRP for 30 min and stabilized chromogen for 30 min. The plate was analyzed according to manufacturer’s protocol and Aβ42 values were normalized to total protein concentration of lysates. Two independent experiments and cells from 2 HC and AD patients with 3 technical replicates (wells) were performed in this experiment.

### Mice brain perfusion and tissue processing

Mice were anaesthetized with 2.5% (v/v) Avertin (Sigma-Aldrich). Transcardial perfusion with 50 mL cold PBS was performed using a peristaltic pump with the perfusate flow rate not exceeding 10 mL/min. Brain tissue processing was performed as described previously^50,83^. Hemibrains were isolated and fixed in 4% PFA overnight at 4°C before transferring to 30% sucrose in PBS at 4°C for preservation. Hemibrains were cryosectioned coronally at a thickness of 40 μm on a freezing-sliding microtome, and the free-floating sections were stored in cryoprotectant (40% PBS, 30% glycerol, 30% ethylene glycol) and kept at −20°C until staining.

### Immunofluorescence and image acquisition and analysis

Human adult fibroblasts were fixed with 4% PFA for 15 min at room temperature, permeabilized with 0.3% Triton X-100 in PBS for 5 min or cold Methanol on ice for 20 min, blocked with pre-warmed 5% BSA in PBS and shook for 45 min at room temperature. Fibroblasts were incubated with primary antibody solution in 5% BSA overnight at 4°C in a moisture chamber: anti-CHMP2B (1:400; Proteintech, 12527–1-AP), anti-Galectin-3 (1:1000; Biolegend, 125401), anti-γ-H2AX (1:200; Millipore Sigma, 05–636), anti-H3K9me3 (1:500; Abcam, ab8898), anti-H4K16ac (1:300; Thermo Fisher Scientific, MA5–27794), anti-LAMP1 (1:2000; Cell Signaling, 9091), anti-LAMP2 (1:200; DSHB, H4B4), anti-p62/SQSTM1 (1:400; Abcam, ab56416), anti-S100A4 (1:200; Abcam, ab124805), anti-Ubiquitin (1:200; LifeSensors, AB120), and anti-Vimentin (1:400; Cell Signaling, 5741). Fibroblasts were washed with PBS three times and incubated with fluorophore-conjugated secondary antibody solution in the dark for 1 hr at room temperature: anti-mouse, anti-rabbit and anti-rat (1:500, Thermo Fisher Scientific). Finally, fibroblasts were washed with PBS three times, mounted with DAPI containing ProLong Glass Antifade Mountant (Thermo Fisher Scientific) and air-dried for overnight prior to imaging.

Human tNeurons were undergone two-step fixation: 1) removed half of the culture medium and added equivalent to half the volume of 4% PFA for 5 min at room temperature; 2) removed the old solution and added 4% PFA for 15 min at room temperature. The tNeurons were permeabilized with 0.3% Triton X-100 in PBS for 5 min at room temperature or cold Methanol on ice for 20 min, blocked with pre-warmed 5% BSA in PBS and shook for 45 min at room temperature. tNeurons were incubated with primary antibody solution in 5% BSA overnight at 4°C in a moisture chamber: anti-ASC/PYCARD (1:100; Santa Cruz, sc-514414), anti-GAP-43 (1:300; Novus Biologicals, NB300–143), anti-MAP2 (1:1000; Biolegend, 822501), anti-NeuN (1:500; Abcam, ab177487), anti-NLRP3 (1:200; R&D systems, MAB7578), anti-p62/SQSTM1 (1:500; Proteintech, 18420–1-AP), anti-pTau (AT8) (1:100; Thermo Fisher Scientific, MN1020), anti-pTau (S262) (1:200; FUJIFILM WAKO, 010–27123), anti-pTDP-43 Ser409/410 (1:300; Cosmo Bio, CAC-TIP-PTD-M01 & 1:200; Biolegend, 829901), anti-Synapsin-1 (Syn-1) (1:200; Abcam, ab64581), anti-Tau (1:100; Aves Labs), and anti-Beta tubulin III (Tuj1) (1:500; Neuromics, CH23005 & 1:1000; Biolegend, 801201). The tNeurons were washed with PBS three times and incubated with fluorophore-conjugated secondary antibody solution in the dark for 1 hr at room temperature: anti-mouse, anti-rabbit, anti-rat, and anti-chicken (1:500, Thermo Fisher Scientific), washed with PBS three times, mounted with DAPI containing ProLong Glass Antifade Mountant (Thermo Fisher Scientific) and air-dried for overnight prior to imaging.

A detailed protocol for immunostaining of free-floating frozen and paraffin-embedded tissue sections can be found at https://dx.doi.org/10.17504/protocols.io.dm6gp356pvzp/v1. Briefly, free-floating mouse tissue sections at 30 to 50 μm were collected and proceed for immunostaining in multi-well plates. Mouse tissue sections were first rinsed with PBS three times and permeabilized with 0.3% Triton X-100 in PBS for 20 min at room temperature, followed by incubation with blocking buffer (10% Normal Donkey Serum and 0.03% Triton-X-100 in PBS) and shook for 1 hr at room temperature. After PBS rinsing, the tissue sections were incubated with primary antibody solution in 10% Normal Donkey Serum in PBS overnight at 4°C in a moisture chamber: anti-Amyloid-β (1–42) (1:100; Enzo Life Sciences, ADI-905–804-100), anti-CHMP2B (1:100; Proteintech, 12527–1-AP), anti-Galectin-3 (1:50; R&D systems, AF1197), anti-Hsp70 (1:300; Abcam, ab45133), anti-LAMP1 (1:50; Santa Cruz, sc-19992), and anti-MAP2 (1:500; Biolegend, 822501). Fluorophore-conjugated secondary antibody solution was incubated for 1 hr at room temperature: anti-mouse, anti-rabbit, anti-goat and anti-chicken (1:500, Thermo Fisher Scientific). Tissue sections were washed with PBS two times, counterstained with Hoechst (1:2000) and washed with PBS two times again before mounting with ProLong Glass Antifade Mountant (Thermo Fisher Scientific). Samples were air-dried for overnight prior to imaging.

Paraffin-embedded human brain tissues of the cerebral cortex were sectioned at 10 μm thickness. Deparaffinization was achieved by washing slides through xylenes twice, each for 5 min and rehydrating via gradient ethanol (100% → 95% → 70% → 50%) into water, each for 10 min. Following heat-mediated antigen retrieval using Citrate buffer, pH 6.0 (Sigma-Aldrich) for 30 min at 95° C, tissue sections were rinsed with PBS once and incubated with blocking buffer (10% Normal Donkey Serum and 0.03% Triton-X-100 in PBS) for 2 hr at room temperature. Slides were incubated with antibody cocktails in blocking buffer overnight at 4°C in a moisture chamber: Amyloid-β (6E10) (1:100; Biolegend, 803001), anti-CHMP2B (1:100; GeneTex, GTX118181), anti-Galectin-3 (1:50; R&D systems, AF1197 & 1:100; Biolegend, 125401), anti-Hsp70 (1:300; Abcam, ab45133), anti-LAMP2 (1:200; Abcam, ab213294), and anti-MAP2 (1:500; Biolegend, 822501). Tissue sections were incubated with fluorophore-conjugated secondary antibody solution for 1 hr at room temperature: anti-mouse, anti-rabbit, anti-rat, anti-goat and anti-chicken (1:500, Thermo Fisher Scientific). After a PBS rinse, slides were counterstained with Hoechst (1:2000) and washed with PBS two times. The complete air-dried tissue sections were mounted with ProLong Glass Antifade Mountant (Thermo Fisher Scientific) and subjected to dry in the dark overnight prior to imaging.

Imaging was acquired at Z-series (10 to 30 sections; 0.2 to 1 μm steps) according to experimental paradigm using Zeiss LSM 700 confocal fluorescence microscope with 20x, 63x and 100x objectives. In each experiment, all groups were subjected to image using the same acquisition settings. The z-stack images were performed Maximum Intensity Projection to analyze the mean pixel intensity and determine a threshold to quantify puncta number in the cells by Fiji. For quantitative histology, three to five separate sections were sampled using a 20x objective and fluorescence signals were measured from entire image field to the mean fluorescence change. Tissue sections were imaged and analyzed by blinded observers.

### Transmission electron microscopy

Cells were grown on Ibidi dishes: μ-Dish 35 mm, high Grid-50 Glass Bottom is a 35 mm then fixed in Karnovsky’s fixative: 2% Glutaraldehyde (EMS Cat# 16000) and 4% PFA (EMS Cat# 15700) in 0.1 M Sodium Cacodylate (EMS Cat# 12300) pH 7.4 for 1 hr, chilled and sent to Stanford’s CSIF on ice. They were then post-fixed in cold 1% Osmium tetroxide (EMS Cat# 19100) in water and allowed to warm for 2 hr in a hood, washed 3X with ultra-filtered water, then en bloc stained 2 hr in 1% Uranyl Acetate at room temperature. Samples were then dehydrated in a series of ethanol washes for 10 min each at room temperature beginning at 30%, 50%, 70%, 95%, changed to 100% 2X, then Propylene Oxide (PO) for 10 min. Samples were infiltrated with EMbed-812 resin (EMS Cat#14120) mixed 1:1, and 2:1 with PO for 2 hr each. The samples were then placed into EMbed-812 for 2 hr opened then placed into flat molds w/labels and fresh resin and placed into 65°C oven overnight. Cells of interest were located using the grid pattern and cut out with a gem saw and remounted on pre-labeled resin blocks with fresh resin and polymerized overnight again. Once full polymerized the glass coverslip was etched away using hydrofluoric acid for 20 min. Using the finder grid pattern left behind the block faces were trimmed down allowing for serial sectioning of the cells of interest. Sections were taken around 90 nm, picked up on formvar/Carbon coated slot Cu grids, stained for 40seconds in 3.5% Uranyl Acetate in 50% Acetone followed by staining in 0.2% Lead Citrate for 6 min. Observed in the JEOL JEM-1400 120kV and photos were taken using a Gatan Orius 2k × 2k digital camera.

### Cytokine profiling analysis on neuronal conditioned medium using Luminex multiplex analysis

Secretion of inflammatory factors was analyzed using cytokine profiling of the conditioned medium from tNeurons of healthy donors and AD patients as previously described (https://dx.doi.org/10.17504/protocols.io.n2bvj3qm5lk5/v1). Conditioned medium was collected 48 hr after the last medium change in a 12-well plate with 1 mL of neuronal maturation medium at PID 38, and centrifuged at 10,000 × G for 10 min at room temperature to pellet out particulates. For Human 80 plex panel (EMD-Millipore), a minimum of 200 μL of supernatants was stored at −80°C. Cell free medium was also collected to monitor the background fluorescence. Cell numbers were determined by an automated cell counter for normalization of cytokine levels. The setup of cytokine profiling assay was performed according to the manufacturer’s instructions. Briefly, samples were mixed with antibody-linked magnetic beads on a 96-well plate and incubated overnight at 4°C with shaking. Cold and room temperature incubation steps were performed on an orbital shaker at 500 to 600 rpm. Plates were washed twice with wash buffer in a Biotek ELx405 washer. Following one hr incubation at room temperature with biotinylated detection antibody, streptavidin-PE was added for 30 min with shaking. Plates were washed as above and PBS added to wells for reading in the Luminex FlexMap3D Instrument with a lower bound of 50 beads per sample per cytokine. Each sample was measured in duplicate. Custom Assay Chex control beads were purchased from Radix Biosolutions, Georgetown, Texas, and are added to all wells. The analyses of all conditioned medium samples were performed using raw data (mean fluorescence intensity (MFI)) rather than concentration (pg/mL) to avoid calculating bias per as per recommendation of the Stanford Human Immune Monitoring Center.

### CSF samples and protein discovery

We used the SOMAScan assay platform^84,85^ (SomaLogic Inc.) to measure the relative levels of 76 human proteins in CSF. This platform is based on modified single-stranded DNA aptamers (SOMAmer) capable of binding to specific protein targets with high sensitivity and specificity. We collected 79 CSF samples (50 HC and 29 AD samples) from a multi-ethnic cohort of older American adults (age range: 60 to 87 years) between 2015 and 2020. Samples were stored at −80°C and 150 μL aliquots of CSF were sent on dry ice to SomaLogic. CSF samples were analyzed via SOMAScan assay in five batches. To account for variation within and across batches, control, calibrator and buffer samples are added in each 96-well plate. Data normalization was conducted by the manufacturer following three stages. First, in Hybridization Control Normalization, hybridization control probes are used to remove individual sample variance. Second, Intraplate Median Signal Normalization, median normalization removed inter-sample differences within the plate. Last, Plate Scaling and Calibration, this final step removed variance across assay runs.

### Quantification and statistical analysis

Quantification of fluorescence images was performed by CLARIOstar plate reader software and open-source Fiji software. For each technical replicate, the fluorescence intensity of the background from cell-free solution or cell-free area in the image field was subtracted from intensity measurements. For most experiments, a power analysis was performed to pre-determine the sample sizes based on results from initial experiments. For quantifications of cytokine levels and human and mouse brain samples, the experiments were blinded. Statistical analysis was performed with unpaired t-test, one- way ANOVA or two-way ANOVA based on the experimental design using GraphPad Prism Software. All values were expressed as the box-and-whisker plots or mean ± SD. Differences between two groups were analyzed using two-sided unpaired t-test with Welch’s correction. Differences between multiple groups were analyzed using one- way or two-way ANOVA followed by Bonferroni post-hoc analysis. Differences were considered statistically significant for P values < 0.05.

## Figures and Tables

**Fig.1:** Transdifferentiating human adult fibroblasts into neurons reveals signatures of aging and Alzheimer’s disease (AD). **(a)** Experimental layouts for this study. Human adult fibroblasts are collected from donors of healthy young and aged, aged with sporadic AD (aged/sAD) and familial AD with *PSEN1* mutations (fAD-PSEN1). Fibroblasts and the transdifferentiated neurons (tNeurons) are used for molecular and proteomic characterization of aging and AD and tested for cellular responses to small molecules. Validation of cellular findings is performed in post-mortem brain tissue or cerebrospinal fluid (CSF) from AD mouse models and patients. **(b)** Levels of DNA damage measured by increased numbers of the nuclear foci of γ-H2AX immunofluorescence (IF) shown in the representative images of human fibroblasts. *n* = 248 (young), 304 (aged) and 252 (aged/sAD) from three donors and three independent experiments. **(c)** Age- and AD-related epigenetic alterations measured by histone modifications H3K9me3 and H4K16ac IF in human fibroblasts. H3K9me3: *n* = 252 (young), 241 (aged) and 237 (aged/sAD) from four donors and three independent experiments. H4K16ac: *n* = 157 (young), 167 (aged) and 141 (aged/sAD) from four donors and three independent experiments. **(d)** Representative images of human fibroblasts immunostained for S100A4 and Vimentin, and tNeurons immunostained for Tuj1, GAP43, MAP2 and NeuN with DAPI counterstaining at post-induction day (PID) 35. Scale bar: 100 μm. **(e)** IF quantification of DNA damage in tNeurons revealed by numbers of the nuclear foci of γ-H2AX. *n* = 150 (young), 132 (aged) and 141 (aged/sAD) from three donors and three independent experiments. **(f)** IF quantification of changes in histone modifications H3K9me3 and H4K16ac in tNeurons. H3K9me3: *n* = 95 (young), 123 (aged) and 124 (aged/sAD) from three donors and three independent experiments. H4K16ac: *n* = 112 (young), 115 (aged) and 117 (aged/sAD) from three donors and three independent experiments. **(g)** Representative images and IF quantification of proteostasis- and disease-associated protein markers in tNeurons, including autophagy adaptor p62/SQSTM1, ubiquitin, Aβ42, hyperphosphorylated tau (pTau) and TDP-43. Cyan dash line outlines tNeuron morphology determined by Tuj1 staining. White dash line represents the nuclear region (N). Scale bar: 20 μm. Data show box-and-whisker plots with 5th, 25th, 50, 75th and 95th percentiles. Differences between groups are compared with One-Way ANOVA followed by Bonferroni post-hoc analysis. **P < 0.01 and ***P < 0.001. See also Extended Data Fig.2c, Fig.3a and Table 1.

**Fig.2:** Human tNeurons carry proteomic signatures of aging and AD. **(a)** Differential expression of proteins detected in tNeurons from healthy young (*n* = 3) and aged (*n* = 3) individuals, and patients with aged/sAD (*n* = 6) at PID 40. The top pathways for aging and sAD proteomes are analyzed using GO databases. Comparison is performed between tNeurons from aged and young donors, and between aged/sAD and aged donors. Colored circles represent the enrichment of identified proteins revealing by log2-fold change: increase in red and decrease in blue. **(b)** List of differentially expressed proteins associated with the risk genes for age-related neurodegenerative diseases. AD: Alzheimer’s disease. PD: Parkinson’s disease. ALS/FTD: Amyotrophic lateral sclerosis/Frontotemporal dementia. **(c)** Clustering heatmap of Pearson correlation coefficients of total tNeuron protein expression. Cluster A to M show distinct protein expression patterns and the associated GO terms between young, age and aged/sAD. Each line represents the expression of individual protein defined by the relative protein abundance (z-score) across different groups. White circles connecting with the black lines representing the average z-score for each cluster. Dash lines representing ±SD. See also Extended Data Fig.4 and Extended Data Table 2.

**Fig.3:** Deficits in lysosomal repair drive lysosomal dysfunction and Aβ pathology in AD tNeurons. **a)** Transmission electron microscopy (TEM) for analyzing ultrastructural morphology of lysosomes and mitochondria in human tNeurons. Changes in lysosomal size, electron-dense material abundance and mitochondria-lysosome contacts defined by the contact length are detected in tNeurons. Arrowhead: electron-dense materials adjacent to or within lysosomes. Scale bar (i): 20 μm. Scale bar (ii-iv): 1 μm. Lysosomal size: *n* = 59 (young), 69 (aged) and 69 (aged/sAD) from two donors and two independent experiments; electron-dense material abundance: *n* = 74 (young), 68 (aged) and 60 (aged/sAD) from two donors and two independent experiments; mitochondria-lysosome contacts: *n* = 174 (young), 262 (aged) and 246 (aged/sAD) from two donors and two independent experiments. **(b)** The experimental pipeline for testing how aging and AD alters the lysosomal homeostasis and damage response, leading to cell death. **(c)** Representative images of tNeurons immunostained for LAMP2 (green), ESCRT-III CHMP2B and Galectin-3 (magenta) and Tuj1 (blue) at PID 35 at basal conditions. IF quantification of numbers of CHMP2B and Galectin-3 puncta in the cell body of tNeurons without the treatment of any lysosomal damage agent. CHMP2B: *n* = 117 (young), 103 (aged) and 97 (aged/sAD) from three donors and three independent experiments; Galectin-3: *n* = 111 (young), 108 (aged) and 95 (aged/sAD) from three donors and three independent experiments. Insert: higher magnification view of CHMP2B and Galectin-3 colocalization with LAMP2. Scale bar: 10 μm. **(d)** Comparison of numbers of CHMP2B and Galectin-3 puncta between fibroblasts and tNeurons at basal conditions. Fibroblasts: CHMP2B: *n* = 102 (young), 105 (aged) and 99 (aged/sAD); Galectin-3: *n* = 102 (young), 105 (aged) and 99 (aged/sAD). tNeurons: CHMP2B: *n* = 117 (young), 103 (aged) and 97 (aged/sAD); Galectin-3: *n* = 111 (young), 108 (aged) and 95 (aged/sAD). All data are acquired from three donors and three independent experiments. **(e)** Representative images of AD tNeurons immunostained for LAMP2 (green), ESCRT-0 HGS protein (magenta) and Tuj1 (blue) at PID 36. Cells are treated with a lysosomotropic agent, L-leucyl-L-leucine O-methyl ester (LLOME), at 0.25 mM for 30 min, and then washed out of LLOME for lysosomal repair. IF quantification of numbers of HGS puncta in the cell body during LLOME treatment and washout for up to 8 hr. The half-life (t*1*_*/2*_) represents the time required for lysosomal repair. *n* = 90 to 108 (young), 109 to 116 (aged) and 79 to 117 (aged/sAD) from three donors and three independent experiments. Insert: higher magnification view of HGS and LAMP2 in the cell body and neurites. Arrowhead: HGS colocalization with LAMP2. Scale bar: 10 μm. **(f)** Schematic for describing an experimental pipeline to test if defective LQC mediates mitochondrial dysfunction in AD. Quantification of mitochondrial membrane potential in tNeurons using TMRE staining after the treatment of DMSO Ctrl, 20 μM FCCP or 0.25 mM LLOME for 30 min. The values are revealed by a fold change relative to young tNeurons treated with DMSO. *n* = 6 (young), 6 (aged) and 6 (aged/sAD) from three donors and two independent experiments. **(g)** IF analysis of colocalization of Aβ42 with LAMP1 in aged/sAD tNeurons. Insert: higher magnification view of Aβ42 and LAMP1. Scale bar: 10 μm. **(h)** Analysis of correlation between intra-cellular Aβ42 level and Galectin-3 puncta number in different groups of tNeurons. *n* = 9 from three donors and three independent experiments. Black solid line represents the fitted linear correlation. Coefficient of Discrimination (R^2^) is calculated using Pearson’s correlation. Data show mean and SD or box-and-whisker plots. Differences between groups are compared with One-Way or Two-Way ANOVA followed by Bonferroni post-hoc analysis. *P < 0.05, **P < 0.01 and ***P < 0.001. ###P < 0.001. See also Extended Data Fig.6 and 8.

**Fig.4:** Lysosomal damage is exacerbated in AD and linked to amyloid accumulation in postmortem brain tissue. **(a)** Schematic for describing an experimental pipeline to test the disease phenotypes observed in AD tNeurons are also detected in brain tissue of AD patients and transgenic mice expressing mutant human APP with the Swedish (K670N/M671L) and London (V717I) mutations (APP^Lon/Swe^) for modeling AD. HC: healthy control. **(b)** IF staining of CHMP2B, Aβ42 and LAMP1 in the neocortex of non-transgenic mice (NTg) and APP^Lon/Swe^ transgenic mice. The brain tissue is co-stained with MAP2. Insert: higher magnification view of colocalization between CHMP2B, Aβ42, LAMP1 within MAP2. Arrowhead: intra-neuronal colocalization of CHPM2B and Aβ42 with LAMP1. Scale bar: 10 μm. **(c)** IF quantification of CHMP2B, Aβ(6E10) and LAMP2 in the post-mortem cerebral cortex of HC and AD donors. The brain tissue is co-stained with MAP2 and Hoechst. Insert: higher magnification view of CHMP2B, and Aβ(6E10) colocalization with LAMP2. Arrowhead: CHMP2B-positive fibril structures. Scale bar: 20 μm. See also Extended Data Fig.10 and 11.

**Fig.5:** Lysosomal damage mediates inflammatory responses in AD tNeurons. **(a)** Interaction network for proteins involved in the inflammatory response pathway in tNeurons. The relative abundance indicated by log2-fold change between aged/sAD and aged and between fAD-PSEN1 and aged/sAD tNeurons is shown by color: increase in red and decrease in blue. **(b)** Schematic for describing an experimental pipeline to test if lysosomal damage enhances inflammasome activation and cytokine secretion in AD neurons and if there is a small molecule that reduces lysosomal damage and cytokine secretion. **(c)** Representative images of tNeurons immunostained for inflammasome markers NLRP3 (cyan) and PYCARD/ASC (magenta) with or without 0.25 mM LLOME treatment for 3 hr at PID 40. IF quantification of the percentage of tNeurons showing inflammasomes per image. *n* = 441 to 477 (young), 399 to 513 (aged) and 522 to 648 (aged/sAD) from four donors and three independent experiments. Arrowhead: colocalization of NLRP3 and PYCARD/ASC. Scale bar: 10 μm. **(d)** Experimental schematic for inflammatory profiling of the conditioned medium from all group of tNeurons at basal conditions at PID 40. Heatmap represents the fold changes in cytokine and chemokine levels relative to young tNeurons. *n* = 6 (young), 6 (aged), 12 (aged/sAD) and 4 (fAD-PSEN1) from two independent experiments. Exact log2-fold change (Log2FC) and P values can be found in Extended Data Fig.12a. **(e)** Schematic and heatmap for inflammatory profile of the conditioned medium from young tNeurons treated with or without chronic lysosomal damage stress (0.1 mM LLOME starting at PID 33 for 7 days). The values are revealed by a fold change relative to young tNeurons treated with DMSO. *n* = 6 (young) and 8 (young + LLOME) from two independent experiments. Exact log2-fold change (Log2FC) and P values can be found in Extended Data Fig.12a. **(f)** Heatmap and Pearson correlation analysis for identified cytokines and chemokines of tNeurons at the basal and chronic lysosomal damage conditions from panel (D) and (E). Number in each cell indicates the correlation coefficient. **(g)** Schematic and heatmap for inflammatory profile of the conditioned medium from aged/sAD and fAD-PSEN1 tNeurons at PID 35 with or without the treatment of 3.1 μM C381 for 7 days. The values are revealed by a fold change relative to tNeurons treated with DMSO. *n* = 4 (aged/sAD: vehicle, C381) and 4 (fAD-PSEN1: vehicle, C381) from two independent experiments. Exact Log2FC and P values can be found in Extended Data Fig.12b. Log2-fold change in mean fluorescence intensity (MFI) is used for comparison. Differences between groups are compared with One-Way ANOVA followed by Bonferroni post-hoc analysis. *P < 0.05 and ***P < 0.001. See also Extended Data Fig.12.

**Fig.6:** Pharmacological rescue of lysosomal resilience to damage ameliorates AD phenotypes in fibroblasts and tNeurons. **(a)** Schematic for describing an experimental pipeline to test if there are small molecules that promote LQC to reduce lysosomal damage and provide neuroprotective effects on lysosomal dysfunction, protein accumulation and cell death in AD tNeurons. **(b)** 2D Histogram represents the changes of lysosomal damage in aged, aged/sAD and fAD-PSEN1 tNeurons by 0.25 mM LLOME treatment for 30 min at PID 35 following the pre-treatment with 3.1 μM C381 for 7 days. Ctrl: *n* = 103 (aged), 95 (aged/sAD) and 104 (fAD-PSEN1) from three donors and three independent experiments. C381: *n* = 88 (aged), 95 (aged/sAD) and 91 (fAD-PSEN1) from three donors and three independent experiments. Each dot represents a single neuron and the number of detectable CHMP2B (x-axis) and Galectin-3 (y-axis) puncta in the cell body. **(c)** Measurement of lysosomal proteolysis in tNeurons reflected by Cathepsin-B activity. The changes in Cathepsin-B activity caused by 0.25 mM LLOME treatment for 30 min at PID 35 following the pre-treatment with 3.1 μM C381 for 7 days. *n* = 142 to 147 (young), 122 to 157 (aged), 152 to 157 (aged/sAD) and 141 to 142 (fAD-PSEN1) from three donors and three independent experiments. **(d)** Measurement of Caspase-3/7 activation, a canonical upstream marker of apoptosis, in tNeurons with 0.5 mM LLOME treatment for 1 hr at PID 42. The pre-treatment of 3.1 μM C381 is continued for 7 days. *n* = 105 to 161 (young), 133 to 158 (aged), 142 to 167 (aged/sAD) and 136 to 148 (fAD-PSEN1) from three donors and three independent experiments. **(e)** Schematic for small molecules that modulate lysosomal function and damage. C381: regulates lysosomal pH by targeting v-ATPases. Thioperamide: elevates the levels of bis(monoacylglycero)phosphate (BMP). NCT-504: enhances ESCRT-mediated degradation. LLOME: triggers lysosomal membrane permeabilization. BAPTA-AM: cell-permeable selective Ca2+ chelator that blocks Ca2+-dependent recruitment of ESCRT-III/Vps4 complex to the damaged lysosome. Small molecules with beneficial effects labelled with blue color, whereas with detrimental effects labelled with black color. **(f)** Effects of small molecules (LLOME: 0.25 mM, BAPTA-AM: 2.5 μM, C381: 3.1 μM, Thioperamide: 5 μM and NCT-504: 2.5 μM; 2-day treatment) on intra-neuronal Aβ42 levels in aged, aged/sAD and fAD-PSEN1 tNeurons. Representative images of aged/sAD tNeurons immunostained for Aβ42 (magenta) and Tuj1 (green) at PID 35. IF quantification of fold-changes in Aβ42 levels during small molecule treatment relative to DMSO Ctrl. *n* = 104 to 122 (aged), 88 to 144 (aged/sAD) and 61 to 136 (fAD-PSEN1) from three donors and three independent experiments. Scale bar: 50 μm. **(g)** Summary of aging and AD signatures in fibroblasts and tNeurons, including AD-related deficits in proteostasis and LQC function. Proposed model for the link of defective lysosomal repair and overwhelming LQC system, leading to constitutive lysosomal damage, aberrant protein accumulations, inflammatory responses, and neurotoxicity in AD. The strategy for restoring lysosomal homeostasis and damage ameliorate AD pathologies in neurons. Data show box-and-whisker plots with 5th, 25th, 50, 75th and 95th percentiles. Differences between groups are compared with unpaired t test, One-Way or Two-Way ANOVA followed by Bonferroni post-hoc analysis. **P < 0.01 and ***P < 0.001.
